# Teneligliptin enhances the beneficial effects of GLP-1 in endothelial cells exposed to hyperglycemic conditions

**DOI:** 10.18632/oncotarget.22849

**Published:** 2017-12-01

**Authors:** Valeria De Nigris, Francesco Prattichizzo, Elettra Mancuso, Rosangela Spiga, Gemma Pujadas, Antonio Ceriello

**Affiliations:** ^1^ Institut d’Investigacions Biomèdiques August Pi i Sunyer (IDIBAPS), Barcelona, Spain; ^2^ Istituto di Ricovero e Cura a Carattere Scientifico (IRCCS) MultiMedica, Milan, Italy; ^3^ Department of Medical and Surgical Sciences, University Magna Grǽcia of Catanzaro, Catanzaro, Italy; ^4^ Centro de Investigación Biomédica en Red de Diabetes y Enfermedades Metabólicas Asociadas (CIBERDEM), Madrid, Spain

**Keywords:** GLP-1, DPP-4i, teneligliptin, CVD, high glucose

## Abstract

High-glucose-induced oxidative stress contributes to cardiovascular endothelial damage in diabetes. Glucagon-like peptide 1 (GLP-1) is beneficial to endothelial cells, but its effects are diminished when cells are continuously exposed to high glucose. Teneligliptin is a dipeptidyl peptidase-4 (DPP-4) inhibitor that prevents oxidative stress, apoptosis and the metabolic memory effect. We explored the potential additive effects of Teneligliptin and GLP-1 in hyperglycemia-damaged endothelial cells. Human umbilical vein endothelial cells (HUVECs) were exposed to normal-glucose (5 mmol/L) or high-glucose (HG, 25 mmol/L) for 21 days, or to HG for 14 days followed by normal-glucose for 7 days (HM). These cells were continually treated with Teneligliptin 3.0 μmol/L, alone or in combination with an acute dose of GLP-1 50 nmol/L. DPP-4 was upregulated under hyperglycemic conditions, but Teneligliptin reduced DPP-4 expression and activity. Simultaneous Teneligliptin and GLP-1 synergistically increased the antioxidant response and reduced ROS levels in HG- and HM-exposed HUVECs. Concurrent treatment also enhanced cell proliferation, reduced apoptotic gene expression and ameliorated endoplasmic reticulum stress in HG- and HM-exposed HUVECs. Thus, long-term Teneligliptin treatment reduced DPP-4 levels and activity in HUVECs exposed to chronic hyperglycemia. Moreover, Teneligliptin enhanced the beneficial effects of GLP-1 on oxidative stress, proliferation, apoptosis and endoplasmic reticulum homeostasis.

## INTRODUCTION

Type 2 diabetes mellitus (T2DM) is a major chronic illness causing a series of pathological complications that reduce the quality of life. Cardiovascular disease (CVD) is the most important cause of death in diabetic patients [1]. Hyperglycemia, the major factor implicated in CVD in diabetic patients [2], damages the endothelium mainly by overstimulating the production of reactive oxygen species (ROS) [3], thus increasing oxidative stress and fostering endothelial dysfunction (ED). Lowering glucose levels is not sufficient to switch off the self-perpetuating intracellular pro-oxidant process [4], which is the basis of diabetic cardiovascular complications [5]. This pathogenic mechanism can be partly explained by the “metabolic memory,” defined as the perpetuation of vascular damage despite the achievement of improved glycemic control [4].

Glucagon-like peptide 1 (GLP-1) is an incretin hormone used for the treatment of T2DM [6]. Beyond improving glycemic control [6], GLP-1 exerts vascular protective effects [7] by inducing the expression of antioxidant enzymes such as heme-oxygenase 1 (HMOX) or NAD(P)H-1 dehydrogenase quinone (NQO-1) [8]. Additionally, GLP-1 ameliorates hyperglycemia-induced endoplasmic reticulum (ER) stress in endothelial cells (ECs) [9]. However, our group recently demonstrated that hyperglycemia induces “endothelial resistance” to the positive actions of GLP-1 [10]. This phenomenon partially depends on oxidative stress, and has been observed in multiple tissues exposed to the diabetic milieu [11–13].

**Figure 1:**
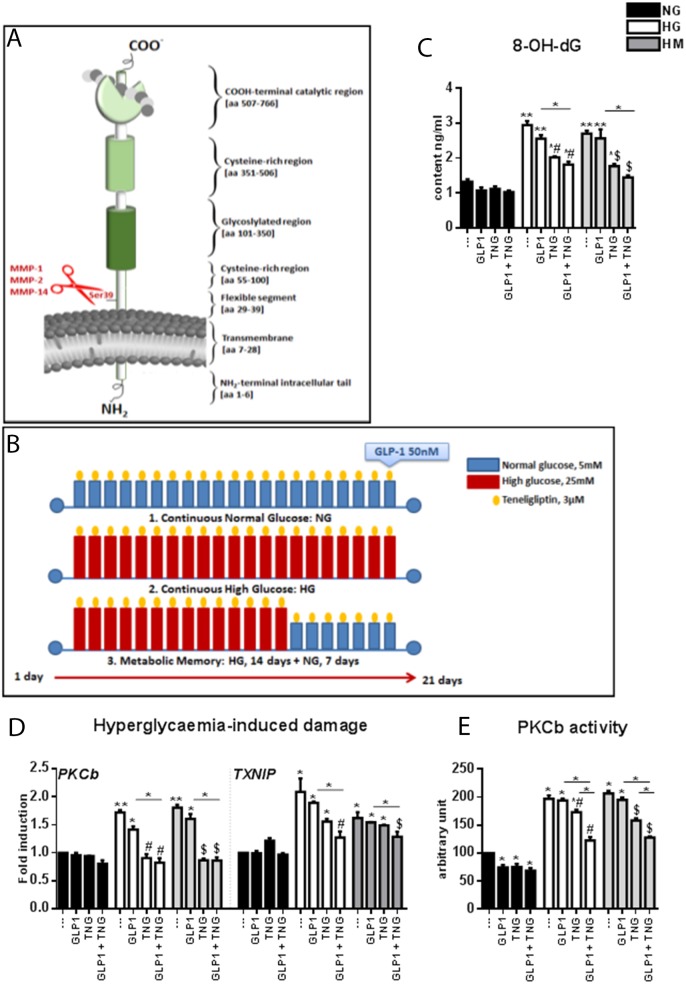
Effects of Teneligliptin, GLP-1 and HG, with or without normalization, on hyperglycemia-induced damage markers and oxidative stress in HUVECs **(A)** Schematic representation of the domain structure of DPP-4. The shedding from the membrane by the indicated MMPs is shown in red. **(B)** Experimental design. Cells were maintained under NG, HG or HM conditions for 21 days. During the exposure, Teneligliptin was continually added to the medium at 3.0 μmol/L. Before cell harvesting, the cells were treated with GLP-1 50 nmol/L for 1 hour. **(C)** 8-OH-dG content (ng/mL) of HUVECs cultured in NG/HG/HM, with or without Teneligliptin and/or GLP-1. **(D)** Markers of hyperglycemia-induced damage in endothelial cells. Total cellular RNA was isolated from HUVECs exposed to the aforementioned conditions, and *PKCβ* and *TXNIP* mRNA levels were assessed by qRT-PCR and expressed relative to *GAPDH*. **(E)** PKCβ activity expressed as arbitrary units (a.u.) in HUVECs cultured under NG/HG/HM conditions, with or without Teneligliptin and/or GLP-1. ^*^p<0.05 and ^**^p<0.01 vs. NG. ^#^p<0.05 vs. HG. ^$^p<0.05 vs. HM. Bars represent the mean±SEM for five (D) or three (C, E) independent experiments. TNG, Teneligliptin.

The inhibition of dipeptidyl peptidase-4 (DPP-4) activity and/or the use of degradation-resistant GLP-1 analogues has been reported to improve endothelial function in clinical settings [14]. DPP-4, also known as CD26, is a 110-kDa type-II transmembrane glycoprotein that can cleave a variety of substrates, including the incretin hormones GLP-1 and gastric inhibitory polypeptide (GIP) [6], from the amino-terminus of a polypeptide with proline or alanine in the second position [15] (Figure 1A). DPP-4 is present on the surface of different cells types [6], but can also be found in the circulation in a soluble form (sDPP-4) [16] upon its cleavage from the cell membrane, a process called “shedding” [17].

DPP-4 inhibitor (DPP-4i) drugs preclude the catalytic activity of DPP-4, which otherwise rapidly inactivates the intestinal hormone GLP-1 [6]. Among DPP-4i drugs, Teneligliptin has recently been commercialized. The efficacy and safety of long-term Teneligliptin monotherapy and combination therapy have been evaluated in T2DM patients [18, 19]. In a previous paper, we demonstrated that Teneligliptin has an intrinsic antioxidant capacity in human umbilical vein endothelial cells (HUVECs) exposed to high-glucose (HG) conditions, as it reduces ROS levels and initiates the transcriptional cascade of antioxidant genes [20]. This DPP-4i also enhances proliferation, reduces apoptosis and improves ER homeostasis under the same stress conditions [20].

In the present study, we explored the possible additive effects of GLP-1 and Teneligliptin on the endothelial response, by examining the antioxidant response, proliferation rate and ER stress in HUVECs exposed to HG conditions.

## RESULTS

### Markers of oxidative stress and hyperglycemia-induced damage are reduced by simultaneous Teneligliptin and GLP-1 treatment

HUVECs were exposed to different glucose conditions: continuous normal glucose (NG: 5 mmol/L) for 21 days, continuous HG (HG: 25 mmol/L) for 21 days, or metabolic memory (HM: continuous HG for 14 days, followed by NG for 7 days). Teneligliptin at 3.0 μmol/L was administered every 48 hours during this time, and GLP-1 was added at 50 nmol/L, alone or in combination with Teneligliptin, 1 hour before cell harvesting (Figure 1B).

Under HG and HM conditions, the levels of 8-hydroxy-2′-deoxyguanosine (8-OH-dG, a marker of oxidative damage) were elevated. These increases were significantly ameliorated when cells were treated with Teneligliptin, but not with GLP-1. The decrease in 8-OH-dG levels was more evident after combined treatment with GLP-1 and DPP-4i, especially in the HM state, although it did not differ significantly from the reduction achieved with Teneligliptin or GLP-1 alone (Figure 1C).

Among the mechanisms proposed to link hyperglycemia with CVD in diabetes, the activation of the protein kinase C (PKC) pathway is particularly important [5, 21, 22]. Exposure to HG and HM increased *PKCβ* mRNA levels (Figure 1D) and PKCβ enzyme activity (Figure 1E). Teneligliptin treatment, but not GLP-1 treatment, reversed these changes. The combination of the DPP-4i + GLP-1 did not further reduce *PKCβ* mRNA levels, but downregulated PKCβ activity further than Teneligliptin alone (Figure 1D and 1E).

The mRNA levels of thioredoxin interacting protein (*TXNIP*), a biomarker of glucose-induced damage [23], were elevated in the HG and HM groups. GLP-1 and Teneligliptin alone did not counteract these increases; only their combination significantly dampened the increases in *TXNIP* (Figure 1D).

### The combination of Teneligliptin and GLP-1 improves the antioxidant response in HUVECs under HG and HM conditions

**Figure 2:**
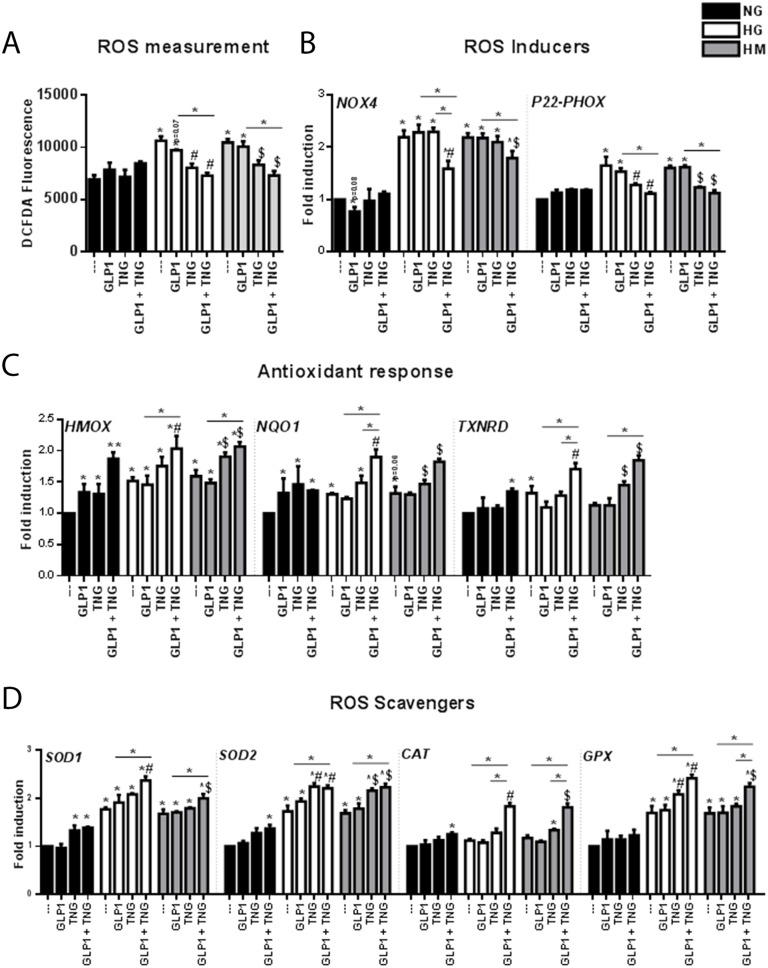
Effects of Teneligliptin and/or GLP-1 on the levels of ROS and pro-oxidant and antioxidant genes in HUVECs cultured under NG, HG and HM conditions **(A)** ROS production, expressed as the levels of the fluorescent product DCFDA, in HUVECs cultured under NG/HG/HM conditions, with or without Teneligliptin and/or GLP-1. **(B, C, D)** Total cellular RNA was isolated from HUVECs, and *NOX4*, *P22^−phox^*, *HMOX*, *NQO1*, *TXNRD*, *SOD1*, *SOD2*, *CAT* and *GPX* mRNA levels were assessed by qRT-PCR and expressed relative to *GAPDH*. ^*^p<0.05 and ^**^p<0.01 vs. NG. ^#^p<0.05 vs. HG. ^$^p<0.05 vs. HM. Bars represent the mean±SEM for five independent experiments. TNG, Teneligliptin.

ROS levels were significantly greater in HG- and HM-exposed cells than in NG-exposed cells (Figure 2A). GLP-1 slightly reduced ROS production in the HG state (^*^p=0.07) but had no apparent effect in the HM state, whereas Teneligliptin reduced ROS levels under both HG and HM conditions. A greater reduction in ROS production was evident after administration of the DPP-4i + GLP-1 (Figure 2A), suggesting that GLP-1 can exert antioxidant effects when it is not rapidly degraded.

To gain insight into the molecular mechanisms underlying this antioxidant effect, we examined the gene expression of the pro-oxidant subunits NOX4 and P22^-phox^ of NAD(P)H oxidase (NOX) [24], one of the molecules involved in ROS production [23]. The mRNA levels of *NOX4* and *P22^-phox^* increased similarly under HG and HM conditions (Figure 2B). Although GLP-1 treatment was expected to reduce the levels of these transcripts under HG and HM conditions, as it did for *NOX4* in the NG state (^*^p=0.08), GLP-1 did not produce these positive effects. On the contrary, Teneligliptin reduced the mRNA levels of *P22^-phox^* but not *NOX4* under both HG and HM conditions. Of note, only the combination of the DPP-4i + GLP-1 reduced the expression of *NOX4* under both hyperglycemic conditions, and further reduced the expression of *P22^-phox^* (Figure 2B).

As nuclear factor (erythroid-derived 2)-like 2 (NRF2) signaling is a major response to ROS overproduction, we analyzed the expression of three NRF2 target genes: *HMOX*, *NQO1* and thioredoxin reductase (*TXNRD*) [25]. In the NG state, Teneligliptin and GLP-1 upregulated the gene expression of *HMOX* and *NQO1*, and their combination further increased the expression of *HMOX*; however, *TXNRD* mRNA levels increased only after simultaneous treatment with the DPP-4i + GLP-1 (Figure 2C). HG and HM treatment increased *HMOX* and *NQO-1* mRNA levels, indicating a possible initiation of the antioxidant cascade. These increases were observed for *TXNRD* only in the HG state. Neither Teneligliptin nor GLP-1 alone exerted positive effects on the expression of these genes in the HG state, whereas Teneligliptin increased *HMOX, NQO-1* and *TXNRD* levels in the HM state. Interestingly, DPP-4i + GLP-1 increased these three antioxidant transcripts under both HG and HM conditions (Figure 2C), and the combination was more efficient than Teneligliptin alone for *NQO-1* and *TXNRD* in the HG state.

Among the endogenous defense systems used by cells to reduce ROS levels, superoxide dismutases (SODs), catalase (CAT) and glutathione peroxidases (GPXs) are important antioxidant enzymes that directly scavenge ROS, converting them into less reactive species [26]. In the NG state, Teneligliptin, but not GLP-1, increased *SOD1* mRNA levels, and the combination of the DPP-4i + GLP-1 upregulated *SOD2* and *CAT* (Figure 2D). HG and HM treatment increased the mRNA levels of *SOD1*, *SOD2* and *GPX*, but did not affect *CAT* levels (Figure 2D). Teneligliptin, but not GLP-1, increased the gene expression of *SOD2* under both HG and HM conditions, and of *GPX* in the HG state. Remarkably, only the combination of the DPP-4i + GLP-1 upregulated all the studied scavengers under both hyperglycemic conditions (Figure 2D).

### The combination of Teneligliptin and GLP-1 improves the proliferative capacity and ER homeostasis of HUVEC cells under HG and HM conditions

**Figure 3:**
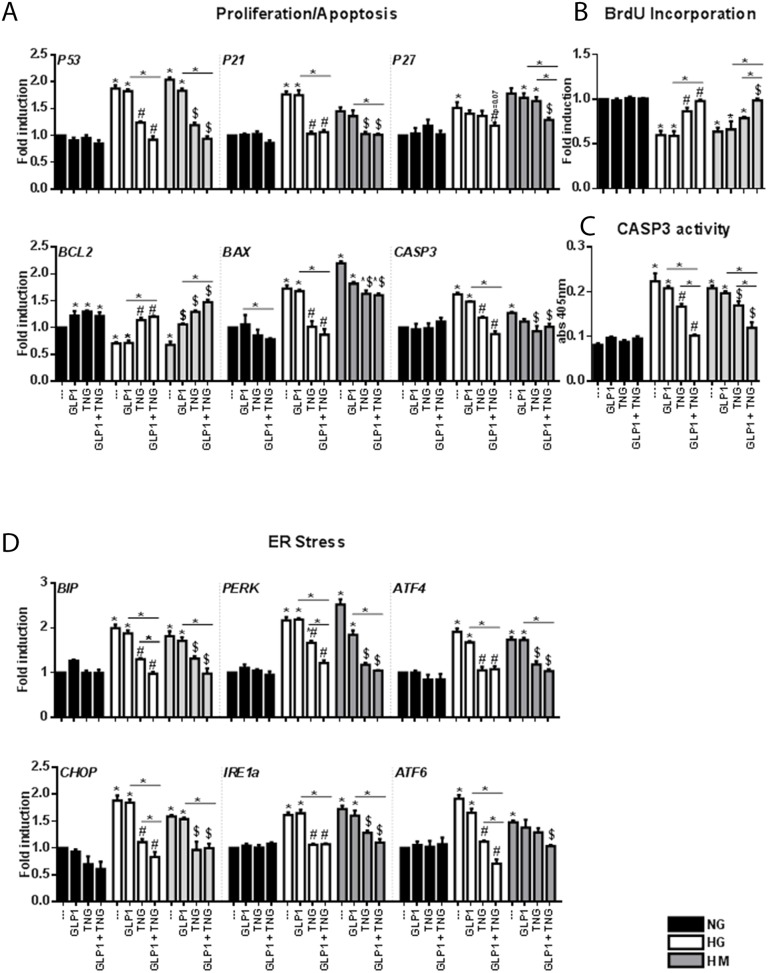
Effects of Teneligliptin and/or GLP-1 on proliferation, apoptosis and ER function in HUVECs cultured under HG/HM conditions **(A, D)** Total cellular RNA was isolated from HUVECs, and *P53*, *P21*, *P27*, *BCL2*, *BAX*, *CASP3*, *BIP*, *PERK*, *ATF4*, *CHOP*, *IRE1a* and *ATF6* mRNA levels were assessed by qRT-PCR and expressed relative to *GAPDH*. **(B)** BrdU incorporation (expressed as fold-induction) and **(C)** caspase activity measured at 405 nm, in HUVECs cultured under NG/HG/HM conditions, with or without Teneligliptin and/or GLP-1. ^*^p<0.05 vs. NG. ^#^p<0.05 vs. HG. ^$^p<0.05 vs. HM. Bars represent the mean±SEM for five (A, D) or three (B, C) independent experiments. TNG, Teneligliptin.

GLP-1 has well-known proliferative properties in the NG state [27], but not under HG conditions [22]. HG and HM treatment of HUVECs increased the gene expression of *P53*, the classical regulator of proliferation pathways. Teneligliptin, but not GLP-1, reduced *P53* mRNA levels, and the combination of the DPP-4i + GLP-1 accentuated this decrease (Figure 3A). *P21* and *P27* mRNA levels increased under HG and HM conditions. Once again, GLP-1 did not counteract these increases, while Teneligliptin, alone or in combination with GLP-1, reduced *P21* mRNA levels under HG and HM conditions. Moreover, only simultaneous administration of the DPP-4i + GLP-1 reduced *P27* mRNA levels in the HM state (Figure 3A).

To confirm these results, we performed a 5-bromodeoxyuridine (BrdU) assay. Teneligliptin treatment, alone or in combination with GLP-1, significantly increased the proliferation rates of cells maintained under HG and HM conditions, while GLP-1 alone did not (Figure 3B).

We next assessed the apoptotic pathway. HG and HM exposure downregulated the anti-apoptotic gene *BCL2*, and upregulated the pro-apoptotic genes *BAX* and *CASP3*. GLP-1 administration increased *BCL2* expression in the NG state, but not under HG and HM conditions. Teneligliptin, alone or in combination with the incretin hormone, increased *BCL2* levels and reduced *BAX* and *CASP3* levels under hyperglycemic conditions (Figure 3A). These results were confirmed with a caspase 3 activity assay (Figure 3C). We also observed that, in the HG state, simultaneous administration of the DPP-4i + GLP-1 had a more pronounced effect on caspase 3 activity than Teneligliptin or GLP-1 treatment alone (Figure 3C).

For their survival, cells must respond to ER perturbations, which are also involved in the pathogenesis of diabetes [23]. We measured the gene expression of several markers of the unfolded protein response, and observed that: (1) *binding immunoglobulin protein* (*BIP*), *protein kinase RNA-like endoplasmic reticulum kinase* (*PERK*), *activating transcription factor 4* and *6* (*ATF4*–*ATF6*), *CCAAT/-enhancer-binding protein homologous protein* (*CHOP*), and *inositol-requiring enzyme 1 alpha* (*IRE1a*) levels increased after HG and HM exposure; (2) Teneligliptin, but not GLP-1, reduced the levels of all the analyzed markers; and (3) the combination of the DPP-4i + GLP-1 further reduced the levels of *BIP*, *PERK*, *CHOP* and *ATF6* in the HG state (Figure 3D).

### Chronic Teneligliptin treatment reduces DPP-4 levels in the medium and *DPP-4* gene/protein levels in HUVECs exposed to HG and HM conditions

Previous studies have demonstrated that plasma DPP-4 activity is elevated in patients with T2DM [28]. This could impair the incretin effect by accelerating the inactivation of GLP-1.

**Figure 4:**
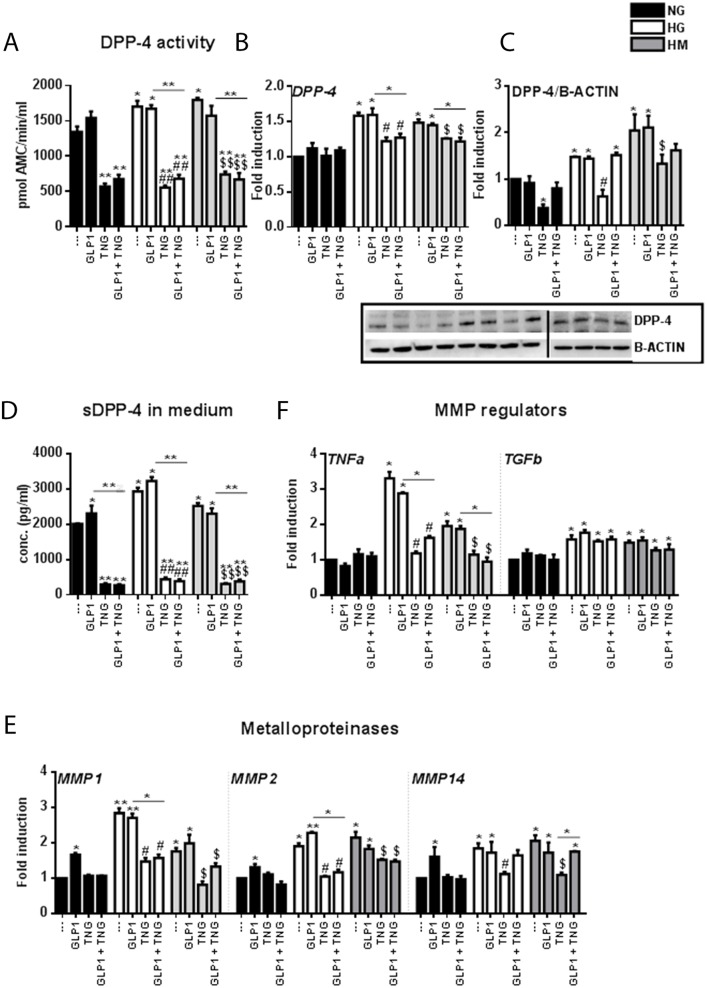
Effects of Teneligliptin and/or GLP-1 on DPP-4 gene expression, protein levels, medium content, activity, and shedding mechanism in HUVECs cultured under HG/HM conditions **(A)** DPP-4 activity in HUVECs cultured under NG/HG/HM conditions, with or without Teneligliptin and/or GLP-1. The results are expressed as the amount of DPP-4 that hydrolyzed the DPP-4 substrate H-Gly-Pro-7-Amino-4-Methyl Coumarin (AMC) to yield 1 μmol of AMC per minute at 37°C. **(B, E, F)** Total cellular RNA was isolated from HUVECs, and the mRNA levels of *DPP-4*, *MMP1*, *MMP2*, *MMP14*, *TNFα* and *TGF*β were assessed by qRT-PCR and expressed relative to *GAPDH*. **(C)** Whole-cell lysates were prepared for Western blot analysis of DPP-4. The panels contain representative images from different independent experiments. Densitometric values were normalized to β-actin. **(D)** DPP-4 content of the culture media of HUVECs exposed to NG/HG/HM, with or without Teneligliptin and/or GLP-1. ^*^p<0.05 and ^**^p<0.01 vs. NG. ^#^p<0.05 and ^##^p<0.01 vs. HG. ^$^p<0.05 and ^$$^p<0.01 vs. HM. Bars represent the mean ± SEM for three independent experiments. TNG, Teneligliptin.

Analysis of DPP-4 activity in HUVECs revealed an increase under HG and HM conditions and a reduction after treatment with Teneligliptin, alone or in combination with GLP-1 (Figure 4A). Moreover, Teneligliptin (alone or in combination with GLP-1) had no effect on *DPP-4* mRNA levels in the NG state, but reduced them under HG and HM conditions (Figure 4B). These results were confirmed by Western blot analysis: HG and HM exposure upregulated DPP-4 protein levels, and Teneligliptin alone strongly attenuated these increases (Figure 4C).

Seeking insight into the effects of Teneligliptin on DPP-4, we quantified DPP-4 levels in the culture medium. Surprisingly, we observed that both HG and HM conditions increased the DPP-4 content of the medium. While GLP-1 did not reverse these increases, Teneligliptin, alone or in combination with the incretin hormone, significantly reduced the DPP-4 content of the culture medium (Figure 4D).

To determine whether Teneligliptin prevents the release of soluble DPP-4, we analyzed the gene expression of matrix metalloproteinases (MMPs) 1, 2 and 14, as these enzymes are involved in the DPP-4 shedding mechanism in smooth muscle cells [29], and the latter two have been found to be elevated in obese mice [30]. In HUVECs, the mRNA levels of all three MMPs increased under HG and HM conditions. GLP-1 did not exert positive effects on these genes, and surprisingly increased their expression in the NG state. On the other hand, Teneligliptin, alone or in combination with GLP-1, attenuated the increases of all three MMPs in the HG state, and of *MMP1* and *MMP2* in the HM state (Figure 4E).

The upstream regulators of MMPs include tumor necrosis factor α (TNFα), which upregulates MMP activity, and transforming growth factor β (TGFβ), which reduces MMP activity [31]. We analyzed the mRNA transcripts of these genes, and found that HG and HM conditions increased *TNFα* and *TGFβ* levels (Figure 4F). Teneligliptin, alone or in combination with GLP-1, reduced *TNFα* but not *TGFβ* mRNA levels, while GLP-1 did not alter the expression of either gene (Figure 4F).

## DISCUSSION

The main findings of the present study are:

  (i) Long-term treatment with Teneligliptin enhances the beneficial effects of GLP-1 in HUVECs exposed to hyperglycemic conditions, by reducing oxidative stress and improving the antioxidant response, proliferation and ER homeostasis.

  (ii) HG and HM conditions cause the enduring upregulation of DPP-4 expression, and Teneligliptin dampens this harmful alteration.

An increase in the 8-OH-dG level is a reliable marker of DNA oxidative damage that persists after glucose normalization [32]. Teneligliptin alone reduced 8-OH-dG formation, and synergistically reduced it in the presence of GLP-1.

We confirmed the direct action of Teneligliptin in restoring ROS levels, increased by HG and HM states in HUVECs. Of note, the combination of the DPP-4i + GLP-1 was more potent than Teneligliptin alone in counteracting ROS production, demonstrating that simultaneous administration of the two drugs powerfully improves the redox state of the endothelium.

The activation of NRF2 is one of the most important cellular mechanisms regulating the expression of phase-II detoxifying enzymes. Oxidative stress stimuli, induced by HG and HM conditions, cause NRF2 accumulation in the nucleus, where it can upregulate the expression of its targets [33]. In our *in vitro* model, GLP-1 alone did not initiate the NRF2-induced antioxidant transcriptional cascade; only simultaneous administration of the DPP-4i + GLP-1 increased the expression of the NRF2 target genes *HMOX*, *NQO-1* and *TXNRD*, and reduced the mRNA levels of *TXNIP*, which is recognized as a connection point in many molecular abnormalities induced by hyperglycemia [34].

ER stress is a major pathological feature induced by hyperglycemia [34], and was not reversed by GLP-1 treatment. Teneligliptin was able to dampen the expression of ER stress markers, and had more pronounced effects when co-administered with GLP-1, indicating that this treatment has both intrinsic effects and synergistic effects with the incretin hormone.

A common cellular outcome linked to hyperglycemia-induced oxidative and ER stress is apoptosis [34]. HG and HM conditions lowered the rate of cellular proliferation and increased apoptosis. The combination of GLP-1 and Teneligliptin restored the pro-proliferative and anti-apoptotic capacities of GLP-1, and even exceeded what the DPP-4i accomplished alone.

The mechanisms regulating the transcription and enzymatic activity of DPP-4 are of interest, but are not yet fully understood. The 5′-flanking region of the *DPP-4* coding sequence has been found to contain DNA elements for gene expression [35]. The factors regulating the expression and tissue distribution of DPP-4 have been studied in several types of cancer, and it has been observed that hypoxia increases the surface levels of DPP-4 [36]. Metformin has also been identified as a previously unrecognized DPP-4i [37], although the mechanisms are not entirely understood and remain controversial.

**Figure 5:**
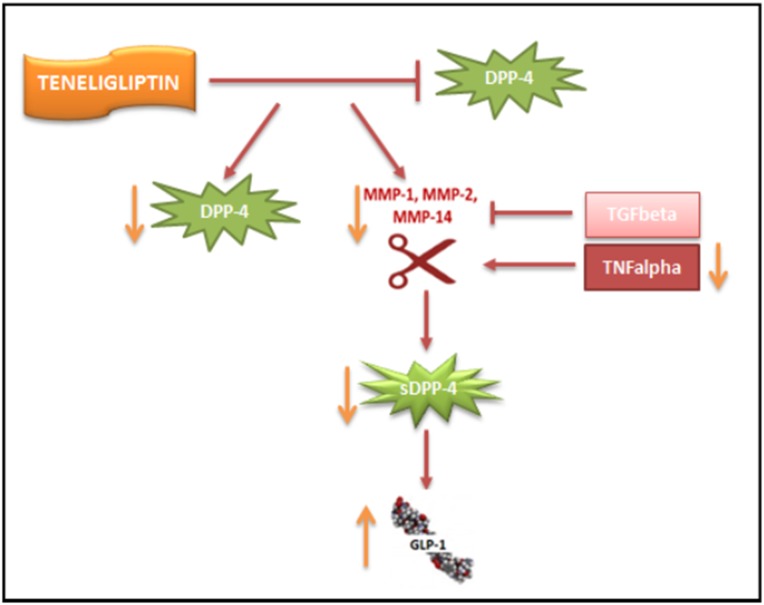
Proposed scheme for the downregulation of DPP-4 by Teneligliptin Teneligliptin downregulates DPP-4 on two different levels: (i) it reduces *DPP-4* mRNA and protein levels under HG conditions; and (ii) it indirectly inhibits the shedding of DPP-4 by downregulating *MMP1*, *MMP2* and *MMP14* gene expression via TNFα.

Here, we have shown for the first time that prolonged treatment with Teneligliptin downregulates DPP-4 activity on two different levels (Figure 5). On the one hand, Teneligliptin can reduce *DPP-4* mRNA and protein levels under HG and HM conditions. In NG-exposed HUVECs, Teneligliptin reduced DPP-4 expression only at the protein level. This can be explained by the fact that Teneligliptin will only downregulate *DPP-4* gene expression in response to an increased amount of DPP-4 protein, which is present only under HG and HM conditions. Interestingly, a recent study on human dermal microvascular endothelial cells (HMVECs) revealed a similar effect on DPP-4 of another DPP-4i, Linagliptin [38]. The study showed that both Sitagliptin and Linagliptin suppressed DPP-4 levels in a cell-free system; however, in TGFβ-treated endothelial cells, only Linagliptin (but not Sitagliptin) suppressed DPP-4 protein levels, demonstrating the differential and intrinsic drug-specific effects of DPP4i drugs [38]. Similarly, Takai et al. reported that Sitagliptin and Linagliptin had similar effects on blood glucose and plasma insulin levels in Zucker diabetic fatty rats; however, DPP-4 activity was significantly lower in the Linagliptin-treated rats than in the Sitagliptin-treated rats, in plasma as well as in vascular tissues [39]. This finding corroborates the hypothesis that, although all DPP-4i drugs have the same function, they may have unique drug-specific effects [38]. On the other hand, Teneligliptin indirectly inhibits the shedding of DPP-4 by downregulating *MMP1*, *MMP2* and *MMP14* gene expression via TNFα under hyperglycemic conditions: a decrease in these metalloproteinases reduces the release of the soluble form of DPP-4 by HUVECs. Consequently, treatment with this specific DPP-4i, by lowering the amount of soluble DDP-4, increases the half-life of GLP-1, which can then exert its positive actions on the vasculature (Figure 5).

Although additional studies are needed to clarify the mechanisms of DPP-4 shedding in the context of diabetes, our results indicate that this DPP-4i could exert positive actions beyond the direct inhibition of the DPP-4 enzyme. This could be of interest in clinical practice, since different molecules of the same class have somewhat different effects on the development of cardiovascular complications [40]. For example, several clinical studies (SAVOR-TIMI53 for Saxaglipitin, EXAMINE for Alogliptin and TECOS for Sitagliptin) demonstrated no cardiovascular benefit of their respective DPP-4i drugs, although cardiovascular safety was not compromised [41–43]. In contrast, the newly published results of the SPEAD-A study, related to the effects of Alogliptin on T2DM patients without CVD history, revealed that Alogliptin reduced the progression of the carotid intima-media thickness in patients [44]. It is important to note that, beyond their differences in clinical settings, certain DPP-4i drugs could have intrinsic properties not strictly linked to their class effects [45].

In conclusion, we have demonstrated that Teneligliptin enhances the beneficial effects of GLP-1 on the antioxidant response, ER function and cellular proliferation. Interestingly, Teneligliptin can reduce the DPP-4 content and activity of HUVECs, thus downregulating DPP-4 expression in a manner reported for only one other DPP-4i, Linagliptin [38].

## MATERIALS AND METHODS

### Cell culture and experimental design

HUVECs were purchased from Lonza and cultured with an EGM™-2 Bulletkit™ (Lonza Ibérica S.A.U., Barcelona, Spain), along with the following supplemental growth factors: Epidermal Growth Factor, Hydrocortisone, human recombinant Fibroblast Growth Factor β, Heparin, 2% Fetal Bovine Serum, and Gentamicin/Amphotericin-B. Cells were cultured in supplemented medium at 37°C in a humidified atmosphere with 5% CO_2_. Cells were used between four and six passages. We seeded cells at an initial concentration of 8×10^4^ cells/well in six-well plates, in order to obtain confluent plates at the end of the experiment and to prevent contact inhibition of cell growth. Twenty-four hours after being seeded, the cells were exposed to three different experimental glucose conditions: continuous normal glucose (NG - 5 mmol/L) for 21 days; continuous high glucose (HG, 25 mmol/L) for 21 days; and metabolic memory (HM - continuous HG for 14 days, followed by NG for 7 days) [20, 46, 47]. Glucose monohydrate was purchased from Sigma-Aldrich (Química, S.L., Madrid, Spain) and was dissolved directly in NG culture medium to produce the HG medium.

Teneligliptin hydrobromide hydrate (3-[(2S,4S)-4-[4-(3-methyl-1-phenyl-1H-pyrazol-5-yl)piperazin-1-yl]pyrrolidin-2-yl-carbonyl]thiazolidinehemipentahydrogenbromide hydrate) was kindly provided by Mitsubishi Tanabe Pharma Corporation (Osaka, Japan). Human GLP-1, fragment 7-37, was purchased from Sigma-Aldrich (Química, S.L., Madrid, Spain). Both Teneligliptin and GLP-1, provided as powders, were dissolved in water and added directly to the culture medium. Teneligliptin at 3.0 μmol/L was administered every 48 hours; GLP-1 was added at 50 nmol/L, alone or in combination with Teneligliptin, 1 hour before cell harvesting. The incubation times were determined in previous studies conducted by our group [20]. HUVECs were cultured for three weeks without being passaged, and the medium was changed every 48 hours. The experimental design is depicted in Figure 1B.

### ROS measurement

The fluorescent probe 2’,7’-Dichlorofluorescein diacetate (H_2_DCFDA) (Sigma-Aldrich Química, S.L., Madrid, Spain), was used to measure the intracellular production of ROS. Approximately 5×10^3^ HUVECs were grown on clear, flat-bottomed, treated 96-well plates for 21 days under NG, HG or HM conditions. At the end of the experiment, the cells were treated with the indicated drugs, and the reactions were stopped by the staining of cells with 20 mM H_2_DCFDA for 30 minutes at 37°C. The intensity of H_2_DCFDA was kinetically measured on a fluorescent microplate reader (Synergy HT, BioTek Instruments, Inc., Winooski, Vermont, USA) in accordance with the manufacturer’s recommendations.

### Oxidative stress marker

The 8-OHdG content of HUVECs was determined with a Bioxytech 8-OHdG-EIA Kit (OXIS Health Products, Portland, OR, USA) in accordance with the manufacturer’s recommendations. The assay was repeated three times, and each sample was run in triplicate.

### PKC kinase activity

PKCβ kinase activity was measured with a PKC Kinase Activity Assay Kit (Abcam, Cambridge, UK) according to the manufacturer’s instructions. HUVECs were cultured in NG, HG or HM conditions for 21 days, with or without Teneligliptin and/or GLP-1, and the medium was changed every 48 hours. At the end of incubation, the cells were lysed, the protein content was measured in Bradford assay buffer (Sigma-Aldrich Química, S.L., Madrid, Spain), and 30 μg of lysate was used to determine PKC-specific kinase activity. The assay was performed in triplicate, and the results are shown as arbitrary units (a.u.).

### RNA isolation and qRT-PCR

Total RNA was isolated from HUVECs with a Total RNA Isolation Kit (Norgen Biotek Corp, Thorold, Ontario, Canada) in accordance with the manufacturer’s instructions. First-strand cDNA was prepared from 1-2 μg of total RNA, the Superscript III RT Kit and random hexamer primers (Invitrogen, Carlsbad, CA, USA) in a total volume of 25 μL, according to the manufacturer’s instructions. The reverse transcription reaction was carried out for 90 minutes at 50°C and for an additional 10 minutes at 55°C. Real-time PCR (qRT-PCR) was performed on an ABI Prism 7900 sequence detection system with SYBR Green reagents (Takara Bio Company, Clontech, Mountain View, CA, USA) and TaqMan® Gene Expression Master Mix (Life Technologies, Madrid, Spain).

### BrdU incorporation

BrdU incorporation was determined with a Cell Proliferation ELISA colorimetric assay (Roche, Mannheim, Germany) according to the manufacturer’s instructions. After sample treatment, cells were labeled overnight with BrdU, and then they were fixed and washed. An anti-BrdU-peroxidase working solution and substrate solution were added, and BrdU incorporation was quantified based on the absorbance at 370 nm in a microplate reader.

### Caspase 3 activity

Caspase 3 activity was measured with a Caspase 3 Colorimetric Assay Kit (Abcam) according to the manufacturer’s instructions. Briefly, after sample treatment, cells were labeled with the substrate DEVD (aspartic acid, glutamic acid, valine, aspartic acid)-p-nitroaniline, and incubated at 37°C for 1 hour. Light emission from the chromophore p-nitroaniline was quantified with a microplate reader at 405 nm.

### Protein extraction

Cells were harvested, and whole-cell lysates were prepared with radioimmunoprecipitation assay buffer (Sigma-Aldrich Química, S.L., Madrid, Spain) containing a protease and phosphatase inhibitor cocktail. The protein content of the lysates was determined with the Bradford reagent.

### Western blot analysis

Protein lysates (30 μg) were resolved by sodium dodecyl sulfate polyacrylamide gel electrophoresis (PAGE-R Gold 4-20% gels, purchased from Lonza Ibérica S.A.U.) and transferred to a polyvinylidene fluoride membrane. The blots were blocked with 5% non-fat dry milk in 20 mM Tris-HCl (pH 7.5), 135 mM NaCl and 0.1% Tween-20, and then were incubated with a monoclonal antibody against human DPP-4 (Abcam, United Kingdom) (1:1000). Human β-actin (1:1000) (Sigma-Aldrich Química, S.L., Madrid, Spain) was used as a loading control. Detection was performed with a secondary peroxidase-linked anti-mouse/rabbit antibody (1:3000) (GE Healthcare Europe GmbH, Barcelona, Spain) and an enhanced chemiluminescence system (Pierce Chemical Co, Rockford, IL, USA), according to the manufacturers’ instructions. Proteins were revealed with a CCD camera (ImageQuantLAS4000, GE Healthcare, UK). The protein content was quantified by computer-assisted densitometry (https://imagej.nih.gov/ij/, ImageJ Software, NIH, USA).

### Human CD26/DPP-4 quantification and activity

The concentration and activity of human CD26/DPP-4 were analyzed in cell culture supernatants by means of a human CD26 ELISA Kit (Thermo Fisher Scientific, USA) and a fluorimetric DPP-4 Activity Assay Kit (Abcam, United Kingdom), respectively, according to the manufacturers’ instructions. The assays were repeated three times, and each sample was processed in triplicate.

### Statistical analysis

All values are represented as the mean ± standard error of the mean (SEM). One-way analysis of variance was performed with GraphPad Prism 5 (GraphPad Software, Inc., La Jolla, CA, USA) to determine the statistical significance of differences among the groups. Two-tailed Student’s *t* tests were used to validate the significance of differences between groups.

## Abbreviations

H_2_DCFDA: 2’, 7’-Dichlorofluorescein diacetate
ATF4 – ATF6: activating transcription factor 4 and 6
BIP: binding immunoglobulin protein
CVD: cardiovascular disease
CHOP: CCAAT/-enhancer-binding protein homologous protein
DDP-4: dipeptidyl peptidase-4
ER: endoplasmic reticulum
ED: endothelial dysfunction
GIP: gastric inhibitory polypeptide
GA: gentamicin/amphotericin
GLP-1: glucagon-like peptide 1
HMOX-1: heme oxygenase-1
HG: high glucose
HM: high metabolic memory
HUVECs: human umbilical vein endothelial cells
IRE1a: inositol-requiring enzyme 1 alpha
NQO-1: NAD(P)H dehydrogenase quinone-1
NOX: NAD(P)H Oxidase
NG: normal glucose
NRF2: nuclear factor (erythroid-derived 2)-like 2
PERK: protein kinase RNA-like endoplasmic reticulum kinase
ROS: reactive oxygen species
TRX: thioredoxin
TXNIP: thioredoxin interacting protein
TXNRD: thioredoxin reductase
T2DM: type 2 diabetes mellitus
UPR: unfolded protein response
